# Source-Dependent Quality Variation in Shoulder Dislocation Videos on YouTube

**DOI:** 10.1016/j.asmr.2024.100921

**Published:** 2024-02-29

**Authors:** Mehmet Kaymakoglu, Taha Aksoy, Ulas Can Kolac, Erdi Ozdemir, Nicholas N. DePhillipo, Gazi Huri, Flippo Familiari

**Affiliations:** a Department of Orthopedics and Traumatology, Faculty of Medicine, Izmir University of Economics, Izmir, Turkey; b Department of Orthopedics and Traumatology, Hacettepe University Faculty of Medicine, Ankara, Turkey; c Department of Orthopaedics & Rehabilitation, Penn State College of Medicine, Hershey, Pennsylvania, U.S.A.; d Department of Orthopedics, University of Pennsylvania, Philadelphia, Pennsylvania, U.S.A.; e Department of Orthopaedics, Magna Graecia University of Catanzaro, Catanzaro, Italy

## Abstract

**Purpose:**

To assess the quality of YouTube videos for patient education on shoulder dislocation.

**Methods:**

A standard YouTube search was performed in March 2023 using the terms “shoulder dislocation,” “dislocated shoulder,” and “glenohumeral joint dislocation” to identify eligible videos. Multiple scoring systems, including DISCERN (a validated tool for analyzing the quality of health information in consumer-targeted videos), Journal of the American Medical Association (JAMA) Benchmark Criteria, and the Global Quality Score (GQS) were used to evaluate the videos. Video quality scores from various sources were compared using the Kruskal-Wallis test for initial analysis, followed by Dunn's post-hoc test with Bonferroni correction, and the strength of relationship between variables was assessed using Spearman's rank correlation coefficient.

**Results:**

A total of 162 eligible videos were identified. The mean video duration was 11.38 ± 3.01 minutes, the median number of views was 653. Median number of days since upload was 1,972, the median view rate was 0.343, and median number of likes was 66.12. Based on the DISCERN classification, a substantial proportion of videos were classified as insufficient quality, with 19.4% as “very insufficient” and 42.1% as “insufficient”; 24.1% were classified as “average” quality, whereas only 13.1% were classified as “good” and 1.2% were “excellent.” Videos from academic and professional sources showed a significant positive correlation with DISCERN scores (rho: +0.784, *P* < .001) and greater scores on all 4 scoring systems compared to health information websites.

**Conclusions:**

This study reveals that the majority of YouTube videos on shoulder dislocation lack sufficient quality for patient education, with content quality significantly influenced by the source.

**Clinical Relevance:**

Examining the accuracy of information that patients encounter on YouTube is essential for health care providers to direct individuals toward more reliable sources of information.

The majority of joint dislocations arise from the shoulder joint.^1^ The shallow glenoid cavity, which limits the contact with the humeral head during movement, is primarily responsible for this instability in the shoulder or glenohumeral joint.^2^ The glenohumeral joint can dislocate as a result of the application of substantial anterior or posterior forces, such as a powerful blow to the shoulder or excessive rotational loading.^3^ The majority of cases of shoulder dislocation are caused by falls, injuries from motor vehicle accidents, and contact sports.^4^ Shoulder dislocation may result in complications such as recurrent joint instability, labral tears, nerve injury, rotator cuff tears, and fractures of proximal humerus and/or the glenoid.^5^

YouTube has substantially increased its popularity in recent years, and now YouTube is the main platform for the distribution and consumption of video content.^6^ YouTube is an important source of both knowledge and entertainment by virtue of its large library of user-generated content and expert productions. For more information on musculoskeletal injuries, such as shoulder dislocations, and its treatment, millions of invididuals consult online resources like YouTube videos.^7^ However, finding medically related informative videos that offer accurate and useful guidance can be challenging resulting from the abundance of videos and unqualified sources that upload videos to YouTube.^8^ Therefore, the purpose of this study is to assess the quality of YouTube videos for patient education on shoulder dislocation. We hypothesized that a significant number of the videos reviewed in this study would be low quality for patient education.

## Methods

In this study, a search of the YouTube database was conducted on March 1, 2023, using Safari (version 16.3) with a cleared cache, cleared cookies, and a personal YouTube account that was not in use. “Shoulder dislocation,” “dislocated shoulder,” and “glenohumeral joint dislocation” search terms were used to qualify the top 100 videos depending the “relevance” assignment of the YouTube algorithm. Consistent with the standard user experience on YouTube, we maintained the default “Relevance” filter for sorting the search results. This decision was made to closely mimic a typical user’s search behavior. The choice of the term “shoulder dislocation” over more technical terms like “glenohumeral instability” was intentional. This approach was adopted to simulate a common user’s search. Specific inclusion criteria for videos were being recorded in the English language, having primary content pertaining to shoulder dislocation, and possessing audiovisual quality that met acceptable standards. Exclusion criteria were videos that were repetitive, consisted of only audio or visual content, were not in the English language, did not pertain to shoulder dislocation, solely focused on physical therapy and rehabilitation, as well as news, drama, and satire videos. The study did not impose any restrictions on the length of the videos, and those with multiple episodes were treated as a single item.

The assessment of the YouTube videos was conducted by experienced orthopaedic consultant (M.K.). For each YouTube video included in the final analysis, data were collected on several features including the title, video duration, number of views, video source/uploader, video content type, number of days since upload, view rate (views per day), and number of likes. For the purpose of this study, video sources and uploaders were categorized based on the nature of the organization or individual responsible for the video. The categories were as follows:

Academic: Videos uploaded by universities, educational institutions, or individuals associated with academic research. These were identified based on the presence of university branding, affiliations mentioned in the video or description, and credentials of the speakers. Health information websites: Videos uploaded by websites or channels primarily dedicated to providing health-related information. These were recognized by their focus on a range of health topics, the absence of direct commercial interests, and the provision of educational content rather than personal or anecdotal experiences. Medical advertising/for-profit companies: Videos uploaded by commercial entities or individuals with a clear commercial intent. Identification was based on explicit branding, promotional content, or calls to action for products or services related to medical treatment.

In addition, video content was classified into several categories based on the primary focus of the video: Description of shoulder dislocation: Videos primarily focused on explaining the anatomy, causes, or general information about shoulder dislocation. Medical treatment: Content predominantly discussing nonsurgical treatment options, including medications, physical therapy, and conservative management techniques. Surgical treatment: Videos focusing on surgical procedures, techniques, and postoperative care specific to shoulder dislocation. Complications of treatment and surgery: Videos that specifically address potential risks, complications, and challenges associated with both surgical and nonsurgical treatments of shoulder dislocation. These classifications were determined by a detailed review of each video’s content, the credentials of the presenter or uploader, and the overall intent and focus of the video as evident from its visual and auditory content as well as the accompanying descriptions.

**Appendix Fig 1:**
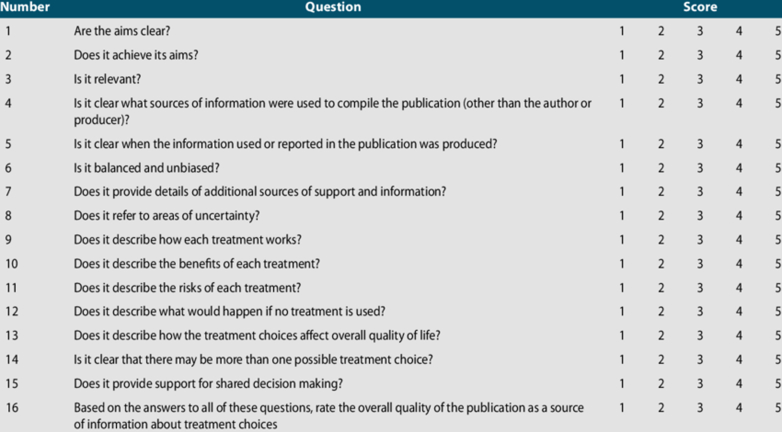
DISCERN instrument.

**Appendix Fig 2:**
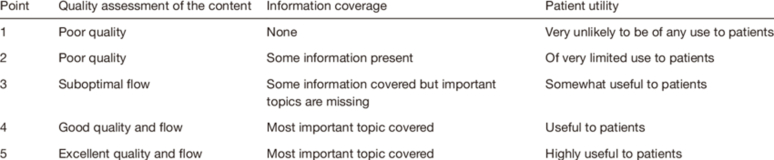
Global Quality Score (GQS).

Authenticity and accuracy of the videos were evaluated by the Journal of the American Medical Association (JAMA) Benchmark Criteria, which assigns one point for each of its 4 criteria: (1) authorship, (2) attribution, (3) disclosure, and (4) currency. A greater JAMA score suggests a greater level of accuracy and dependability.^9^ The DISCERN and Global Quality Score (GQS) scores were used to evaluate the educational value of videos (Appendix Figs 1 and 2, available at www.arthroscopyjournal.org). The GQS uses a 5-point scale, with scores ranging from 1 (“low quality”) to 5 (“high quality”).^10^ The DISCERN score is composed of 16 questions, scored between 1 and 5, with a total score ranging from 16 to 80. Greater scores on the DISCERN scale suggest better quality. In the DISCERN scoring system, videos were rated as “very poor,” “poor,” “fair,” “good,” or “excellent.”^11^ To determine the quality of a video, we considered both technical aspects and content quality. Technical quality included video resolution and sound quality, ensuring that the video was easily viewable and audible. Content quality was assessed based on the accuracy of the information presented, the balance of perspectives, and the depth of coverage on the topic of shoulder dislocation. A “high-quality” video was thus defined as one that scored well on both these technical aspects and content criteria, providing reliable, clear, and comprehensive information on shoulder dislocation suitable for patient education. Adequate information for patient education is defined as content accurately and comprehensively covering shoulder dislocation, understandable to nonmedical audiences, and presenting evidence-based treatment options. A “low-quality source” lacks accuracy, presents biased or incomplete treatment information, and fails in audiovisual clarity, potentially misleading viewers

A single researcher (M.K.) evaluated and scored the videos using the DISCERN, GQS, and JAMA scoring systems. The quality scores assigned using DISCERN, JAMA Benchmark Criteria, and GQS were analyzed to determine whether the type of content and source influenced the educational value of the videos. No ethical approval was required because the study did not involve human subjects or animals.

### Statistical Analysis

Statistical analysis was performed with IBM SPSS Statistics for Windows (version 22, IBM SPSS Corp., Armonk, NY). Continuous data are shown as means and medians, whereas categorical data are represented as frequencies (n) and percentages (%). To compare the quality scores among videos from different sources, we employed the Kruskal-Wallis test, a nonparametric method suitable for our data’s distribution characteristics. This approach enabled us to evaluate differences in medians of DISCERN, JAMA, and GQS scores across the categories of video sources. Significant findings from the Kruskal–Wallis test were further explored using the Dunn test with Bonferroni correction for post-hoc analysis. The strength of relationship between quantitative variables was analyzed by Spearman rank correlation coefficient.

## Results

**Fig 1:**
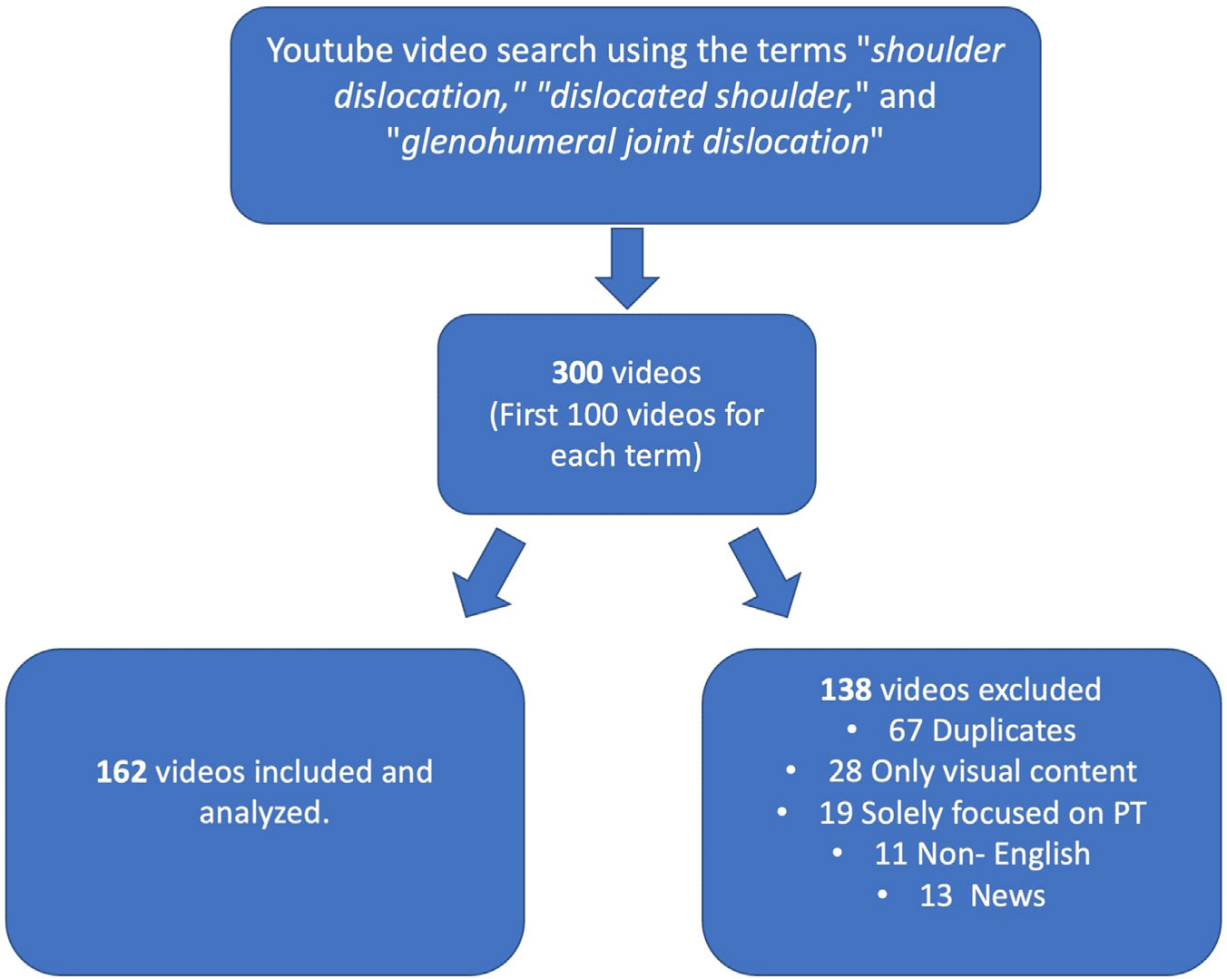
In total, 300 English-language videos on shoulder dislocation were identified from YouTube using the algorithm’s relevancy ranking and meeting specific criteria, after we excluded videos that were repetitive, non-English, news, or solely focused on physical therapy (PT).

A total of 300 videos were identified. After we removed 67 duplicates, 28 videos with purely visual content, 19 videos only focusing on physical therapy, 11 non-English videos, and 13 news were excluded from the analysis. After applying the exclusion criteria, 162 videos were included in the final analysis (Fig 1). The mean video duration was 11.38 ± 3.01 minutes (range 1.41-22.48 minutes), whereas the median number of views was 653 (range 159-4167). The median number of days since upload was 1972 (range 101-5,681), the median view rate was 0.343 (range 0.01-83.25), and the median number of likes was 66.12 (range 0-5,327). Video characteristics are presented in Table 1.

**Table 1:** Video Characteristics of the YouTube Videos

Characteristics	Mean	Standard Deviation	Minimum	Maximum
Video duration, min	11.38	3.01	1.41	22.48
Views	653	221.3	159	4167
Days since upload	1972	873	101	5681
View ratio	0.343	0.034	0.011	83.25
Comments	11.6	14.3	0	83
Likes	66.12	26.8	0	5,327

**Fig 2:**
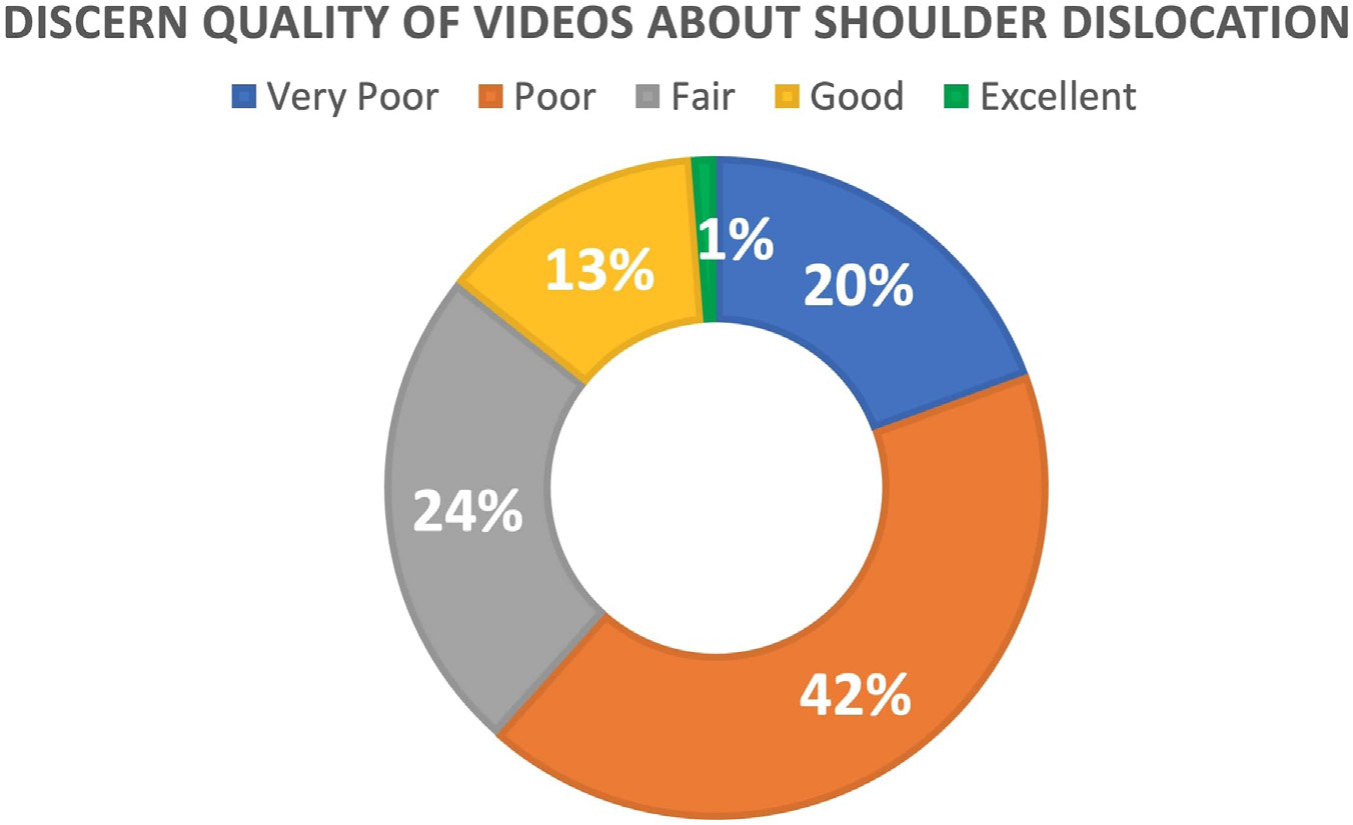
DISCERN scores of YouTube Videos.

According to the DISCERN scoring system, a large proportion of videos were categorized as insufficient in quality, with 19.4% classified as “very insufficient” and 42.1% as “insufficient”; 24.1% were considered as “average,” whereas only 13.1% were rated as “good” and 1.2% as “excellent” (Fig 2). The mean scores for each scoring system are presented in the Table 2. The findings also revealed significant correlations between the DISCERN and GQS, DISCERN, and JAMA scoring systems. Our analysis revealed significant correlations between DISCERN scores, video sources, and content types. Notably, videos from university channels/health professionals were associated with greater DISCERN scores (rho: +0.784, *P* < .001) (Table 3).

**Table 2:** Scores for Each Scoring System

Criteria	Average	SD	Median	Min.	Max.
DISCERN	44.73	10.71	39	18	63
GQS	2.97	0,62	3	1	5
JAMA	2.82	0.49	3	1	4

GQS, Global Quality Score; JAMA, Journal of the American Medical Association Benchmark Criteria.

**Table 3:** Correlations of Quantitative Variables and Scores

	DISCERN (*P* Value)	GQS (*P* Value)	JAMA (*P* Value)
Video duration	.742; .345	**<.001;** **.693**	.841; .431
Video source	**<.001;** .784	**<.001;** **.738**	**<.001;** **.882**
Video content	**<.001;** **.672**	**<.001;** **.642**	.071 .595
Video views	.508; .352	.621; .451	.84; .297
Comments	.231; .396	.653; .457	.531; .538
Likes	.491; .464	.603; .479	.733; .591

NOTE. Values shown are *P* value; rho = Spearman’s rho. Statistically significant *P* values are shown in bold.

GQS, Global Quality Score; JAMA, Journal of the American Medical Association Benchmark Criteria.

The GQS score was significantly correlated with video source (rho: 0.738, *P* < .001), video duration (rho: 0.693, *P* < .001), and video content (rho: 0.642, *P* < .001), and the JAMA score was significantly correlated with video source (rho: 0.882, *P* < .001) (Table 3). The videos sourced from university channels/health professionals had significantly greater scores on all 3 scoring systems (DISCERN, GQS, and JAMA) compared with health information websites (*P* < .05 for all comparisons). Similarly, videos sourced from medical advertisements/for-profit companies had significantly lower scores on DISCERN and GQS (*P* < .05) compared with videos from university channels/health professionals. However, there were no significant differences in the scores of health information websites and medical advertisements/for-profit companies (*P* > .05 for all comparisons) (Table 4). In addition, videos focusing on description of shoulder dislocation received greater scores than the videos that focused on medical or surgical treatments (Table 5).

**Table 4:** Scores for Each Scoring System Per Video Source

Video Source	Number of Videos	DISCERN	GQS	JAMA
University channels/health professionals (doctors, etc.)	98	49.4	3.6	3.3
Health information web sites	41	31.1	2.7	2.4
Medical advertisements/profit companies	23	26.4	2.3	2.4
University channels/health professionals vs health information web sites		**<.001**	**<.001**	**.039**
University channels/health professionals vs medical advertisements/profit companies		**.018**	**<.001**	**.029**
Health information web sites vs medical advertisements/profit companies		.456	.143	**<.001**

NOTE. Statistically significant *P* values are shown in bold.

GQS, Global Quality Score; JAMA, Journal of the American Medical Association Benchmark Criteria.

**Table 5:** Scores for Each Scoring System per Video Content

Video Content	Number of Videos	DISCERN	GQS	JAMA
Description of shoulder dislocation	84	47.4	3.24	3.10
Medical management	34	38.1	2.92	2.83
Surgical management	30	33.6	2.56	2.71
Complications	14	36.1	3.01	3.06

GQS, Global Quality Score; JAMA, Journal of the American Medical Association Benchmark Criteria.

## Discussion

The most important results of this study were that greater than 61% of YouTube videos on shoulder dislocation were of insufficient quality for patient education, and videos sourced from university channels/health professionals were of significantly greater quality content compared with those sourced from medical advertisements/for-profit companies. These findings echo the concerns raised by Sudah et al.,^12^ who found that patient education materials from the American Academy of Orthopaedic Surgeons regarding shoulder conditions often exceed recommended readability levels, indicating a broader issue in the accessibility and quality of health education materials across different mediums. Moreover, similar to Etzel et al.’s assessment of YouTube content on shoulder instability,^13^ our study underscores the importance of evaluating the source of YouTube videos when assessing their quality and usefulness in patient education. The significance of source credibility, as highlighted by our findings, reinforces the need for health care providers to guide patients toward more reliable sources of health information.

YouTube has grown exponentially as a result of increased access to digital platforms and become more important for consumer learning over time. In particular, the number of medical videos on YouTube is rapidly expanding, as viewers often turn to YouTube to find information on health-related topics or to better understand specific medical procedures.^14^^,^^15^

In selecting YouTube as the sole platform for our analysis, we recognized its status as the most widely used video-sharing platform globally, especially for educational content. YouTube’s extensive reach and established role as a primary source of health information for patients and professionals alike guided our decision. Its unique combination of widespread accessibility, user engagement, and content diversity makes it a critical site for evaluating the quality of health-related video content. Moreover, YouTube videos often appear prominently in search engine results, further influencing public access to health information. Although emerging platforms like TikTok are gaining popularity and may offer health-related content, they typically feature shorter videos and are geared more toward entertainment rather than detailed educational material.^16^ This feature is supported by Bethell et al.’s evaluation^16^ of TikTok content related to shoulder stability exercises, which illustrates the challenge social media platforms face in providing accurate and useful health education.

Recognizing YouTube as an effective communication tool, companies and hospitals have increased their production of video content.^17^ Video materials are published, especially in areas like shoulder dislocation, to raise awareness among patients and health care professionals. Despite the increasing prevalence of YouTube as a resource of medical knowledge, there remains a notable shortage of literature specifically addressing the quality of YouTube videos related to shoulder dislocation. This gap highlights the pressing need for additional research efforts to comprehensively evaluate and enhance the reliability and educational value of online resources pertaining to shoulder dislocation.^18^

This study aimed to evaluate the quality of YouTube videos related to shoulder dislocation and revealed that of the 162 videos analyzed, only 23 (14.3%) received high scores across all evaluation criteria, indicating a need for improvement in the quality of videos related to shoulder dislocation. This finding aligns with a previous study conducted by Cassidy et al.,^18^ which similarly reported that the most of the videos on YouTube concerning anterior cruciate ligament injury and therapy are lacking sufficient educational quality. The consistency in results across studies emphasizes the urgent need for interventions to enhance the eligibility and credibility of educational content available on online platforms like YouTube.^19^ Moreover, our study revealed that videos uploaded by academic sources obtained the greatest scores in terms of quality and reliability. Conversely, videos uploaded by medical advertising or profit-oriented companies were associated with the lowest scores, indicating a discrepancy in the value and reliableness of information provided by different sources (Table 4). These findings are consistent with the study of Baker et al.,^20^ further supporting the notion that the credibility and trustworthiness of videos on medical topics are influenced by the source of the video. The discrepancy in content quality, particularly the lower scores associated with videos uploaded by medical advertising or profit-oriented companies, highlights a universal concern regarding the trustworthiness of health information on digital platforms. This concern is shared by Matzko et al.,^21^ who found similar issues with the quality of YouTube videos on SLAP tears. The overall evidence suggests the importance of considering the source and underlying motivations when assessing video content.^20^

This study highlights that YouTube should be used with caution, especially for patients looking for information on shoulder dislocation. Patients should look for videos from trusted sources to ensure that the information is accurate and credible.^22^ The current study also emphasizes the significance of teaching patients how to recognize trustworthy sources of information and the necessity for stricter regulation and quality control of health-related content on YouTube.^23^ YouTube is growing rapidly, with more than 300 videos uploaded per minute and more than 100 million video views per day.^24^ Unfortunately, most of the medical content on YouTube is unreliable, and videos uploaded here are not subjected to quality control or peer review. This is disquieting when it comes to patients seeking medical information, as they may be exposed to potentially harmful or incorrect information, which may also negatively impact their health outcomes.^25^ In considering the effect of video quality on patient education, it’s crucial to discuss the potential implications of poor-quality videos on patient outcomes, particularly regarding glenohumeral dislocation. Misinformation or incomplete information in such videos can lead to misunderstandings about the severity of the condition, the necessity for timely medical intervention, and the appropriate steps for rehabilitation. Patients might delay seeking professional medical advice, opt for inappropriate self-treatment methods, or have unrealistic expectations about recovery timelines and outcomes. Moreover, inadequate or misleading content could contribute to anxiety, decreased patient satisfaction, and create a lack of trust in health care providers, should the patient's experience not align with the information portrayed in these videos. This is particularly concerning for conditions like glenohumeral dislocations, where the management and rehabilitation processes are complex and highly individualized. Misinformed patients may engage in activities that exacerbate their condition or fail to adhere to prescribed treatment plans, potentially leading to chronic instability, increased risk of reinjury, or prolonged recovery periods. Our study identifies varying quality levels among YouTube videos on shoulder dislocation but does not establish a direct link between video quality and patient outcomes or satisfaction with glenohumeral instability. The impact of video quality on actual health outcomes remains an area ripe for future investigation. This distinction is crucial, as our findings suggest the importance of quality information but stop short of correlating it with clinical results or patient satisfaction.

For orthopaedic surgeons, it’s crucial to be aware of the standards of the sources and material that patients view, especially on websites like YouTube. To improve patient comprehension and assist efficient communication, orthopaedic surgeons should integrate visual demonstrations, such as videos and photos, into their daily practice. The integration of visual aids, including videos and slides, into orthopaedic practice is crucial for optimizing patient understanding and engagement in health care decision-making. Patients may be encouraged to seek accurate information from reliable sources for increasing patient satisfaction and improve treatment outcomes. It’s important to prioritize patient education on identifying reliable sources of information as well as advocating for greater regulation and quality control of health-related content on YouTube.

### Limitations

This study is not without limitations. First, only English-language videos were analyzed, which limits the generalizability of the findings. Second, there may be other factors of video quality that were not taken into consideration because the evaluation criteria used in the study were based on the subjective judgments of a single observer. The analysis focused exclusively on the YouTube website, and only one time point was used for the study. In evaluating YouTube videos on shoulder dislocation, we employed the DISCERN instrument, JAMA, and the GQS, with each highlighting different facets of information quality. Although DISCERN offers depth in assessing treatment information’s clarity and balance, it may not fully capture the effectiveness of visual communication in videos. The JAMA criteria lend credibility by evaluating authorship and sources, but like DISCERN, might not address the unique educational dynamics of video content. GQS provides an overall utility perspective but can oversimplify complex information quality aspects. These tools collectively reveal the nuanced challenge of ensuring accessible, accurate, and engaging patient education through video. However, their varied focus points to an inherent limitation in fully capturing the educational value of video content on complex medical topics like shoulder dislocation. In addition, although the scoring systems employed in the research lacked validation, they were among the most commonly used ones in existing literature. Moreover, the study only focused on the content of the videos and comments or user interactions were not evaluated which may also impact the credibility of the information presented.

## Conclusions

This study reveals that the majority of YouTube videos on shoulder dislocation lack sufficient quality for patient education, with content quality significantly influenced by the source.

## Disclosures

This work received open access funding from Università degli Studi Magna Graecia di Catanzaro within the CRUI-CARE Agreement. All authors (M.K., T.A., U.C.K., E.O., N.N.D., F.F., G.H.) report no conflicts of interest in the authorship and publication of this article. Full ICMJE author disclosure forms are available for this article online, as supplementary material.

## Supplementary Data

ICMJE author disclosure forms
