# Supplementary material for: Source-Dependent Quality Variation in Shoulder Dislocation Videos on YouTube
Source: Arthrosc Sports Med Rehabil. 2024 Feb 29;6(3):100921. doi: 10.1016/j.asmr.2024.100921 (PMC11240016; doi:10.1016/j.asmr.2024.100921)
Supplement: ICMJE author disclosure forms [file mmc1.docx]

**Declaration of interests**
 
☒ The authors declare that they have no known competing financial interests or personal relationships that could have appeared to influence the work reported in this paper.
 
☐ The authors declare the following financial interests/personal relationships which may be considered as potential competing interests:

**Author: Mehmet Kaymakoglu**

Date: 13.10.2023

**Declaration of interests**
 
☒ The authors declare that they have no known competing financial interests or personal relationships that could have appeared to influence the work reported in this paper.
 
☐ The authors declare the following financial interests/personal relationships which may be considered as potential competing interests:

**Author: Taha Aksoy**

Date: 13.10.2023

**Declaration of interests**
 
☒ The authors declare that they have no known competing financial interests or personal relationships that could have appeared to influence the work reported in this paper.
 
☐ The authors declare the following financial interests/personal relationships which may be considered as potential competing interests:

**Author: Ulas Can Kolac**

Date: 13.10.2023

**Declaration of interests**
 
☒ The authors declare that they have no known competing financial interests or personal relationships that could have appeared to influence the work reported in this paper.
 
☐ The authors declare the following financial interests/personal relationships which may be considered as potential competing interests:

**Author: Erdi Ozdemir**

Date: 13.10.2023

**Declaration of interests**
 
☒ The authors declare that they have no known competing financial interests or personal relationships that could have appeared to influence the work reported in this paper.
 
☐ The authors declare the following financial interests/personal relationships which may be considered as potential competing interests:

**Author: Nicholas N. DePhillipo**

Date: 13.10.2023

**Declaration of interests**
 
☒ The authors declare that they have no known competing financial interests or personal relationships that could have appeared to influence the work reported in this paper.
 
☐ The authors declare the following financial interests/personal relationships which may be considered as potential competing interests:

**Author: Filippo Familiari**

Date: 13.10.2023

 
 
 

**Declaration of interests**
 
☒ The authors declare that they have no known competing financial interests or personal relationships that could have appeared to influence the work reported in this paper.
 
☐ The authors declare the following financial interests/personal relationships which may be considered as potential competing interests:

**Author: Gazi Huri**

Date: 13.10.2023
